# Obituary

**Published:** 1910-03-26

**Authors:** 


### OBITUARY.

We have to record the death at Kettering of Mr. James John Roughton, J.P., at the age of 86. Mr. Roughton studied at King's College and became a member of the R.C.S. and L.S.A. in 1846.

The death is reported at Great Warley, Essex, of Mr. Robert Debenham, at the age of seventy-six. He took his M.R.C.S. and L.S.A. in 1856, and was at one time house surgeon at the London Hospital.

The death is announced at Louth of Dr. Thomas James Higgins, L.R.C.S.E. He took his L.R.C.S. Edin. in 1865, his L.M. in the same year, and the M.D. of the Royal University of Ireland in 1868. He was honorary consulting surgeon to Louth Hospital, surgeon to the G.N.R., and was a J.P. for the county of Lincoln.

We have to announce the death, at the age of eighty-two, of Dr. John R. Hughes, of Denbigh. He took his M.D. (Edinburgh) with honours in 1851, and was senior surgeon to the Denbighshire Infirmary and coroner for West Denbighshire until he died. At one time he was president of the North Wales British Medical Association, and contributed papers on sanitary measures and on diphtheria.

The death is reported at Iford Lodge, Market Drayton, Shropshire, of Dr. Folliott James Sandford, V.D., Honorary Surgeon-Major of the 2nd Volunteer Battalion K.S.L.I., at the age of 92. He took his M.R.C.S. in 1846, his L.S.A. in 1847, and his M.D. at St. Andrews in 1862. He had been assistant surgeon to St. Giles' Infirmary, London, M.O.H. to Drayton Union R.D.C., and was consulting physician to Market Drayton Cottage Hospital.

The death is announced at Fareham, Hants, of Dr. William Grafton Curgenven, M.D., at the age of sixty-eight. He became an M.D. of St. Andrews in 1862, an M.R.C.S. of England in 1864, and had studied at the Middlesex, where at one time he held the post of house surgeon. Among his other appointments were the house surgeonship of the Derbyshire General Infirmary and the posts of surgeon and consulting surgeon to Derbyshire Royal Infirmary.

Mr. Cecil Edgar Fish, B.A., M.B., B.C. Camb., M.R.C.S., L.R.C.P. Lond., of the Vale of Clwyd Sanatorium, Ruthin, North Wales, has died at West Worthing. He took his diploma in 1893 and his Cambridge degrees in Medicine and Surgery the year following. He studied at Cambridge, St. Thomas's, and Berlin, and had been house physician at the City of London Hospital for Diseases of the Chest, and house surgeon at the Children's Hospital, Shadwell. He had written a paper on the Nordrach system of treating consumption in the *Midland Medical Journal*.

